# Clinical evidence of growth hormone for infertile women with diminished ovarian reserve undergoing IVF: a systematic review and meta-analysis

**DOI:** 10.3389/fendo.2023.1215755

**Published:** 2023-11-07

**Authors:** Guangyao Lin, Xiufang Zhong, Shengnan Li, Lianwei Xu

**Affiliations:** ^1^ Department of Gynecology, Longhua Hospital, Shanghai University of Traditional Chinese Medicine, Shanghai, China; ^2^ Department of Reproductive Center, Shuguang Hospital Affiliated to Shanghai University of Traditional Chinese Medicine, Shanghai, China

**Keywords:** diminished ovarian reserve, growth hormone, infertility, *in vitro* fertilization, meta-analysis

## Abstract

**Objective:**

To appraise the current randomized clinical trials (RCTs) for evidence of the association of growth hormone (GH) with improved outcomes in infertile women with diminished ovarian reserve (DOR) undergoing *in vitro* fertilization (IVF).

**Methods:**

Relevant RCTs published in Chinese or English were identified through a comprehensive search of nine databases from the period of database inception to April 20, 2023. We included trials investigating adjuvant GH during ovarian stimulation and reported the subsequent outcomes. The group with adjuvant GH treatment and the group without adjuvant GH treatment were set up as the trial and control groups, respectively. The quality of RCTs was measured according to the Cochrane Collaboration Handbook.

**Results:**

Of the 579 studies initially identified, 10 RCTs comprising 852 infertile women with DOR were included. The GH dose of individual trials ranged between 3 and 5 IU/day. Overall, we judged the trials to be at high risk of bias in the blinding domain. Pooled results showed that GH was associated with an increased clinical pregnancy rate (RR = 1.63, 95%CI [1.31, 2.03], *p* < 0.0001) and a greater number of oocytes retrieved (MD = 0.91, 95%CI [0.47, 1.35], *p* < 0.0001). Favorable associations were also observed when ovarian stimulation was combined with GH therapy for improving the optimal embryos rate (RR = 1.84, 95%CI [1.30, 2.59], *p* = 0.0005) and the number of optimal embryos (MD = 0.28, 95%CI [0.08, 0.48], *p* = 0.005) along with reducing the cycle cancellation rate (RR = 0.46, 95%CI [0.24, 0.89], *p* = 0.02). Moreover, GH resulted in an increase in the fertilization rate (RR = 1.33, 95%CI [1.18, 1.50], *p* < 0.00001) and the embryo implantation rate (RR = 1.56, 95%CI [1.21, 2.01], *p* = 0.0006). In addition, there was a significant enhancement in estradiol levels (SMD = 1.18, 95%CI [0.46, 1.91], *p* = 0.001) and endometrial thickness (MD = 0.75, 95%CI [0.41, 1.09], *p* < 0.0001) on the day of hCG. With regard to the total number of days and total dose of gonadotrophins used, GH treatment was correlated with shorter days (MD = -0.26, 95%CI [-0.46, -0.06], *p* = 0.01) and lower dose (MD = -460.97, 95%CI [-617.20, -304.73], *p* < 0.00001) of gonadotrophins applied during ovarian stimulation. Furthermore, GH in conjunction with the GnRH antagonist protocol was more conducive to improving the number of oocytes retrieved when compared with the GnRH agonist protocol (*p* < 0.0001). Moreover, a notable association was also seen in IVF combined with GH more than or equal to 4.5 IU/day to increase the number of optimal embryos and estradiol levels on the day of hCG (*p* < 0.05).

**Conclusion:**

For infertile women with DOR undergoing IVF, adjuvant treatment with GH during ovarian stimulation protocols showed better clinical outcomes, shorter days and lower dosages of gonadotrophin required. Furthermore, well-designed RCTs are needed to verify our results in the future.

**Systematic review registration:**

https://www.crd.york.ac.uk PROSPERO (CRD42023421739)

## 1 Introduction

Diminished ovarian reserve (DOR) is one of the leading problems affecting women’s reproductive health. It is a clinical syndrome characterized by reduced anti-Müllerian hormone (AMH) levels, elevated follicle-stimulating hormone (FSH) levels, and decreased antral follicle count (AFC). DOR is also highly associated with low fertility and even infertility due to the concomitant decrease in oocyte quality with quantity (1, 2). Therefore, assisted reproductive technology (ART) is often considered for infertile women with DOR. Statistics derived from 181,536 ART cycles showed that the incidence of DOR was approximately 19% to 26% in the US ART population (3). Women with DOR are closely correlated with a reduced probability of pregnancy in ART as well (4). Meanwhile, the negative consequences of DOR include a higher incidence of preeclampsia (5), elevated odds of recurrent pregnancy loss (6), and a higher risk of miscarriage (7). An increase in the prevalence of infertility caused by DOR has tremendously influenced quality of life in women. Although various adjuvant reagents and stimulation protocols have been applied to promote outcomes in infertile women with DOR undergoing IVF, effective treatment remains a clinical challenge.

Growth hormone (GH), secreted by the pituitary gland, is involved in metabolism, cell growth, and development (8). Growing evidence in the literature demonstrated the expression of GH in female oocytes, ovarian granulosa, placenta, and uterus, which reveals that GH may play a distinct role in women’s reproduction (9, 10). Clinically, a decrease in GH levels tends to correlate with poor ovarian response, low oocyte quality and cleavage rate in IVF (11). The beneficial role of GH supplementation during ovarian stimulation may facilitate the implantation process by improving endometrial receptivity and promoting the maturation process of luteinization through regulating the number of receptors in granulosa cells of patients with DOR, such as bone morphogenetic hormone receptor, FSH receptor, and LH receptor (11, 12). Recently, the number of randomized controlled trials (RCTs) of GH for infertile patients with DOR receiving IVF has been increasing. However, several clinical studies have yielded controversial results. For example, Shi et al. (13) reported that the GH co-treatment with IVF was related to promoting the clinical pregnancy rate but not to reducing the dose and the duration of gonadotropin application, which was contrary to Zhao’s study (14). Furthermore, Kang et al. (15) confirmed that a greater number of oocytes were retrieved in the GH group compared to the control group. Still, the optimal embryos rate between the two groups illustrated no difference. While Zhang et al. (16) demonstrated that though the number of optimal embryos was improved by GH adjuvant therapy, the number of oocytes retrieved was not enhanced. Therefore, this meta-analysis of RCTs was conducted to inform clinical practice. The study’s concerns were as follows: (1) Does GH improve the outcomes in infertile women with DOR undergoing IVF?; (2) Is the GH co-treatment associated with a reduction in gonadotropin required during ovarian stimulation?

## 2 Materials and methods

We conducted this study according to the preferred reporting items for systematic review and meta-analysis (PRISMA) statement (17) and registered on PROSPERO (registration number: CRD42023421739)

### 2.1 Search strategy and study selection

We systematically searched nine databases, namely, Web of Science, Sinomed, EBSCO, Scopus, PubMed, China National Knowledge Infrastructure (CNKI), Cochrane Library, Wanfang, and VIP Information, from the database inception to April 20, 2023. The search strategy was conducted using the following three components: clinical condition (infertility, diminished ovarian reserve, decreased ovarian reserve, declined ovarian reserve), intervention (growth hormone, *in vitro* fertilization, assisted reproductive technology, intracytoplasmic sperm injection, intrauterine insemination), and study type (randomized clinical trial). No search filters or restrictions were applied. To identify additional papers, we also manually checked the reference lists of the retrieved documents.

Two reviewers (G.Y.L. and X.F.Z.) independently checked the titles, abstracts, and full text of comprehensive searches to establish eligibility. Any ambiguity for inclusion was discussed with the other authors (S.N.L. and L.W.X.).

### 2.2 Inclusion and exclusion criteria

Studies were included if (1) infertility was associated with DOR; (2) DOR was defined as AMH<1.1ng/mL or FSH≥10IU/L or AFC<5~7 follicles (18, 19); (3) infertile women underwent IVF regardless of ovarian stimulation protocol; (4) GH was administered during ovarian stimulation; (5) they were RCTs (including propensity score matching studies); (6) there were no geographical restrictions; (7) the study was published in the Chinese or English language.

Studies were excluded if (1) women had a history of polycystic ovary syndrome, ovarian surgery, endometriosis, or other autoimmune or endocrine dysfunction; (2) the study involved DHEA and other adjuvant treatment; (3) the study was duplicate publication, review, case report, meta-analysis, study protocol, and animal experiment; (4) the full text of the study was not accessible.

### 2.3 Data extraction and quality assessment

Two authors (G.Y.L. and X.F.Z.) independently recorded all articles using standardized forms. The following data were collected: methodological characteristics, study population characteristics, details of the treatments, and outcomes in each group. An ultimate form was generated from the two assessment forms. Two reviewers (G.Y.L. and S.N.L.) independently estimated the quality of the studies based on the Cochrane Collaboration Handbook (http://handbook.cochrane.org), and any disagreements between the reviewers were resolved by discussing with the corresponding author (L.W.X). Each RCT was assigned to six specific domains: sequence generation, allocation concealment, blinding of participants and outcome assessment, incomplete outcome data, selective outcome reporting, and other potential threats. The risk of bias for each domain was rated as low, high, or unclear bias based on the information identified from the included articles.

### 2.4 Statistical analysis

The Revman software 5.3 was adopted to perform statistical analysis. Continuous variables were measured by the mean difference (MD) or standardized mean difference (SMD) with 95% confidence intervals (CIs). Dichotomous results were evaluated by the risk ratio (RR) with 95% CI. *p-*value < 0.05 was considered statistically significant. Additionally, heterogeneity among the studies was reported as I^2^. The random effect model was applied when the heterogeneity was substantial (I^2^ > 50%). Otherwise, the fixed effect model was adopted. To investigate potential sources of heterogeneity, subgroup analysis was applied. Where appropriate and possible, planned subgroup analyses included GH dose (≥4.5 IU/d or <4.5 IU/d) and stimulation protocol (GnRH antagonist protocol or GnRH agonist protocol). A sensitivity analysis was performed to verify the robustness of the results by omitting individual studies.

## 3 Results

### 3.1 Included articles

**Figure 1:**
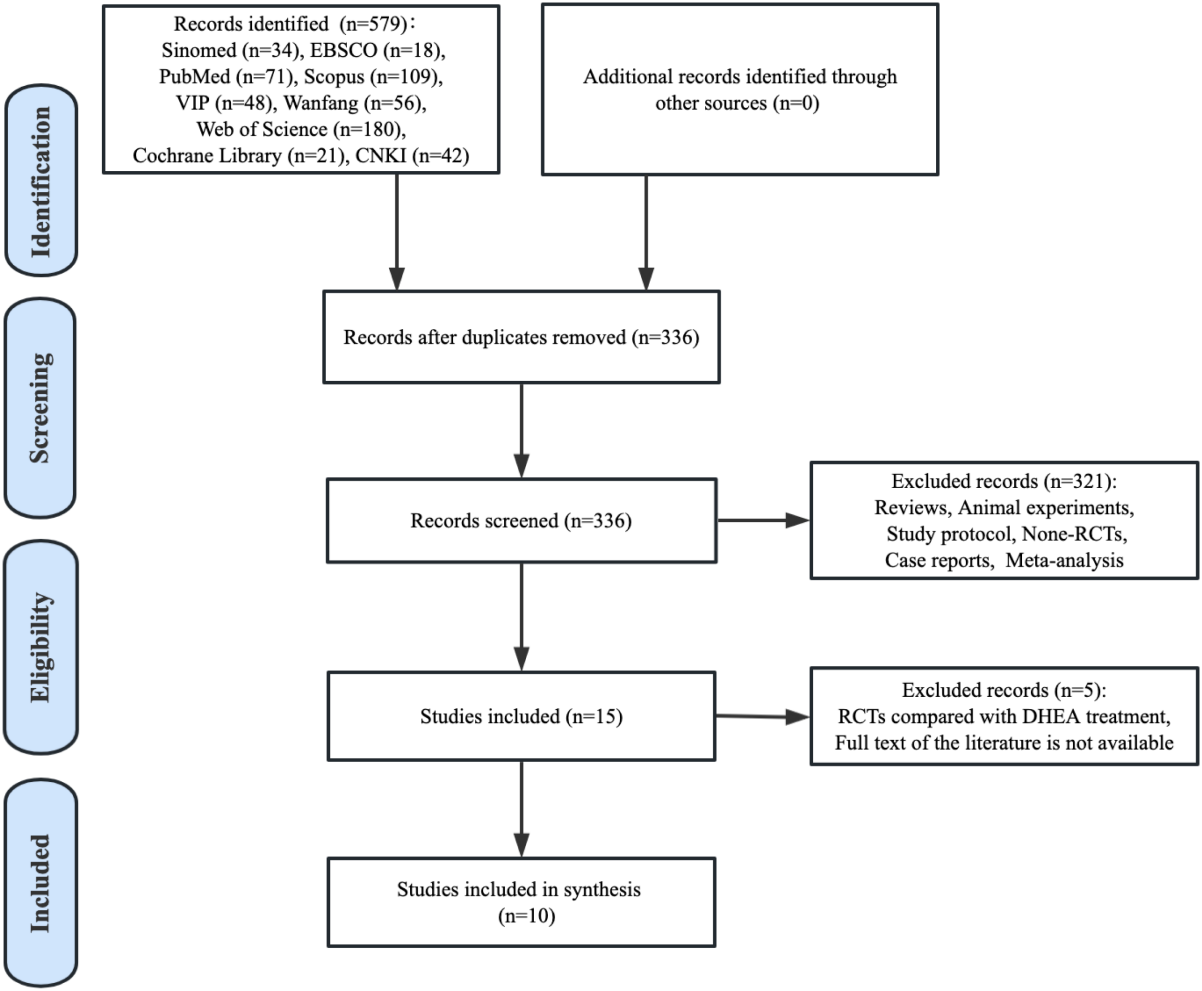
Paper selection flowchart.

Our literature searches yielded 579 potentially relevant citations through database searches, of which 243 duplicates were removed. After screening the titles and abstracts, 321 articles against our inclusion criteria were excluded. Then, after a full-texts review, five publications were excluded as their full texts were not available to access or the studies were compared with DHEA treatment. Finally, 10 RCTs were included for qualitative synthesis (**Figure 1**).

### 3.2 Study characteristics

We included 10 RCTs reporting on 852 infertile women with DOR undergoing IVF. Of these, 427 were divided into the trial group receiving GH co-treatment during ovarian stimulation and 425 into the control group without GH co-treatment. All the included studies were conducted in China and published from 2017 to 2023. Two studies used the median (25th percentile, 75th percentile) for continuous variables. One study (16) stated that the dose of GH used depended on individual AFC development. In addition, one study (20) compared the combination of GH with minimal stimulation; six (13–15, 21–23) compared with GnRH antagonist protocol; one (24) compared with long GnRH agonist protocol; and two (16, 25) compared with short GnRH agonist protocol. Seven trials (13–15, 21–23, 25) applied GH during ovarian stimulation until the trigger day; one study (24) utilized GH during ovarian stimulation for five days and two studies (16, 20) used GH until the leading follicle reached a diameter of 18mm or greater. The study characteristics are presented in **Table 1**.

**Table 1:** Study characteristics.

Study	Year	Sample size (n)	Age (year)		Duration of infertility (year)		BMI (kg/m^2^)		GH dose	Stimulation protocol	Outcomes
		**T/C**	**T**	**C**	**T**	**C**	**T**	**C**			
He (20)	2023	32/32	33 (32, 37)	34 (31, 38)	4.5(2.0,7.3)	2(1.0, 5.3)	20.7(19.9, 21.8)	20.9(19.5, 23.3)	3 IU/d	Minimal stimulation protocol	②⑦⑩
Shi (13)	2022	48/48	36.15 ± 3.02	36.73 ± 2.85	4.08 ± 1.35	4.11 ± 1.37	23.30 ± 2.11	23.31 ± 2.01	4 IU/d	GnRH antagonist protocol	①②③⑥⑦⑧⑨⑩⑪⑫⑬
Wei (21)	2022	24/24	40.36 ± 2.24	40.23 ± 2.18	NA	NA	NA	NA	4 IU/d	GnRH antagonist protocol	②③⑥⑦
Xin (23)	2022	37/37	27.75 ± 2.02	27.93 ± 2.14	4.30 ± 0.49	4.27 ± 0.45	22.06 ± 2.16	22.13 ± 2.01	4 IU/d	GnRH antagonist protocol	①②③⑨⑩⑪⑫⑬
Kang (15)	2020	36/38	39.63 ± 4.85	40.15 ± 4.69	5.17 ± 1.72	5.21 ± 1.70	NA	NA	5 IU/d	GnRH antagonist protocol	①②⑤⑧⑨⑩⑪⑫⑬
Tang (25)	2019	40/42	31.0 ± 3.0	30.0 ± 3.0	4.0 ± 2.5	3.6 ± 2.3	21.7 ± 2.6	22.8 ± 3.6	5 IU/d	Short GnRH agonist protocol	①②⑩⑫⑬
Wang (22)	2018	45/44	38.21 ± 3.95	38.19 ± 3.93	NA	NA	NA	NA	4.51 IU/d	GnRH antagonist protocol	①②③⑥⑦⑩⑫⑬
Zhang (16)	2018	60/60	37.42 ± 1.19	37.91 ± 1.75	5.64 ± 2.80	5.92 ± 2.16	NA	NA	NA	Short GnRH agonist protocol	①②③⑤⑥⑦⑨
Zhao (14)	2017	50/50	38.25 ± 4.52	37.38 ± 4.75	NA	NA	NA	NA	4.5 IU/d	GnRH antagonist protocol	②③⑩⑪⑫⑬
Lin (24)	2017	55/50	36.62 ± 3.17	37.42 ± 3.23	NA	NA	23.31 ± 3.73	23.09 ± 3.79	4.5 IU/d	Long GnRH agonist protocol	①②③⑤⑧⑨⑩⑪⑫⑬

T, trial group; C, control group; BMI, body mass index; GH, growth hormone; NA, not available; GnRH, gonadotropin-releasing hormone; ① Clinical pregnancy rate; ② Number of oocytes retrieved; ③ Optimal embryos rate; ④ Number of optimal embryos; ⑤ Cycle cancellation rate; ⑥ Cleavage rate; ⑦ Fertilization rate; ⑧ Embryo implantation rate; ⑨ Miscarriage rate; ⑩ E_2_ levels on the day of hCG; ⑪ Endometrial thickness on the day of hCG; ⑫ Total days of Gn used; ⑬ Total dose of Gn used.

### 3.3 Risk of bias

**Figure 2:**
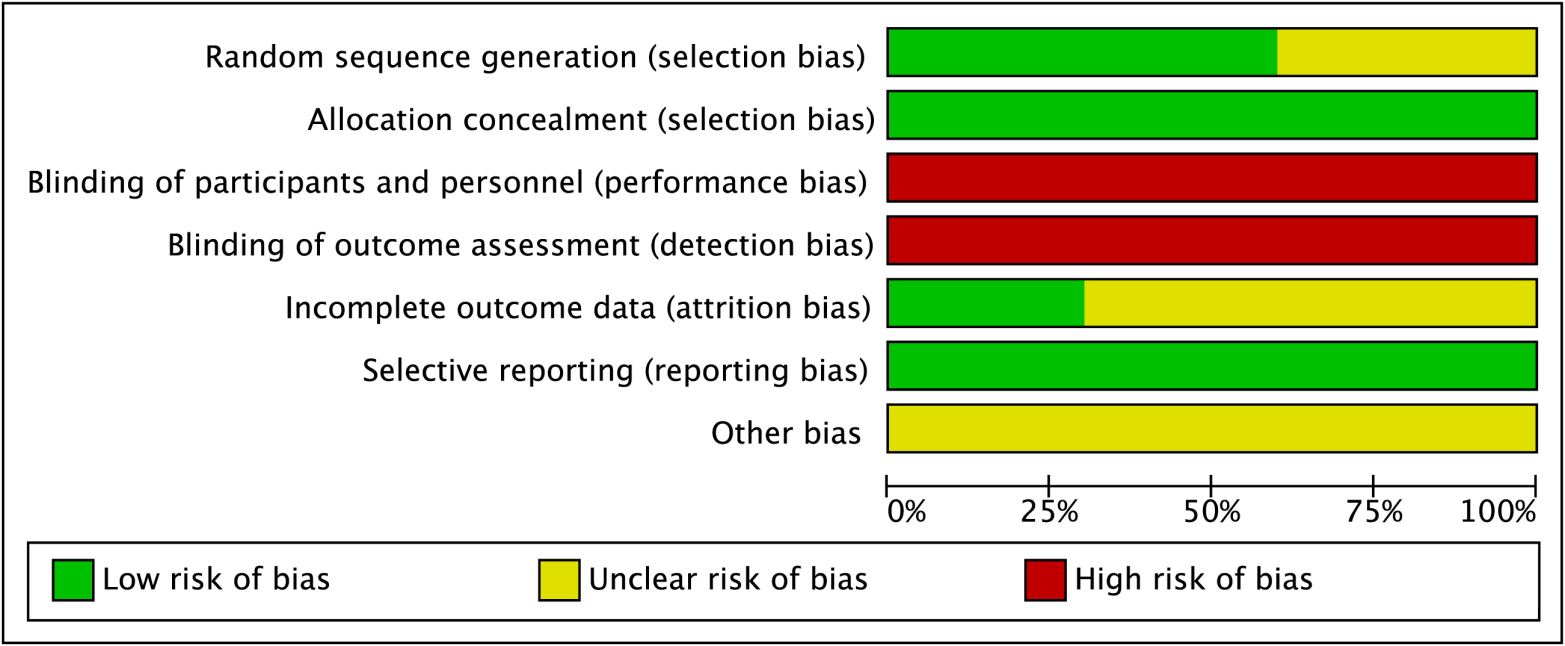
Risk of bias assessment.

The methodological quality of five documents (13, 15, 16, 21, 23) provided detailed procedures on how patients were randomized. Furthermore, one study (20) adopted propensity score matching and was rated as a low risk of bias. Four trials (14, 22, 24, 25) were unclear about random sequence generation. Furthermore, ten studies presented information on the allocation concealment methods, all of which were rated as having a low risk of bias. All studies failed to state the blinding of the participants, investigator, and assessor, which was considered to have a high risk of bias. The incomplete outcome was reported in three studies (15, 16, 24) and was judged as having a low risk of bias. The methods of selected reporting were presented in all studies. No potential bias was detected among the ten studies and, thus, an unclear risk of bias was rated (**Figure 2**).

### 3.4 Outcome measurement

#### 3.4.1 Clinical pregnancy rate and number of oocytes retrieved

Seven RCTs involving 593 women reported clinical pregnancy rates through IVF were meta-analyzed. Pooling the findings of these trials demonstrated that GH co-treatment on IVF significantly improved the clinical pregnancy rate in infertile patients with DOR (RR = 1.63, 95%CI [1.31, 2.03], *p* < 0.0001, I^2^ = 0%). Regarding the number of oocytes retrieved, patients receiving GH achieved a greater number of oocytes retrieved compared with those not receiving GH (MD = 0.91, 95%CI [0.47, 1.35], *p* < 0.0001, I^2^ = 90%) (**Table 2**). The pooled results were robust after checking by sensitivity analysis.

**Table 2:** The pooled results of the forest plot for clinical outcomes.

Clinical outcomes	Study (n)	Case (n)	RR/SMD/MD 95% CI	*p*	I^2^ (%)	Model
Clinical pregnancy rate	7	593	1.63 [1.31, 2.03]	< 0.0001	0	Fixed
Number of oocytes retrieved	8	706	0.91 [0.47, 1.35]	< 0.0001	90	Random
Optimal embryos rate	3	234	1.84 [1.30, 2.59]	0.0005	0	Fixed
Number of optimal embryos	4	375	0.28 [0.08, 0.48]	0.005	94	Random
Cycle cancellation rate	3	299	0.46 [0.24, 0.89]	0.02	0	Fixed
Cleavage rate	4	353	1.12 [0.99, 1.27]	0.07	51	Random
Fertilization rate	5	665	1.33 [1.18, 1.50]	< 0.00001	48	Fixed
Embryo implantation rate	3	430	1.56 [1.21, 2.01]	0.0006	17	Fixed
Miscarriage rate	5	261	1.02 [0.50, 2.10]	0.95	0	Fixed
E_2_ on the day of hCG	7	602	1.18 [0.46, 1.91]	0.001	94	Random
Endometrial thickness on the day of hCG	5	449	0.75 [0.41, 1.09]	< 0.0001	57	Random
Total days of Gn used	5	438	-0.26 [-0.46, -0.06]	0.01	42	Fixed
Total dose of Gn used	6	538	-460.97 [-617.20, -304.73]	< 0.00001	90	Random

Gn, gonadotropin; E_2_, estradiol.

#### 3.4.2 Optimal embryos rate/number of optimal embryos and cycle cancellation rate

It was noteworthy that the rate of optimal embryos was significantly higher (RR = 1.84, 95%CI [1.30, 2.59], *p* = 0.0005, I^2^ = 0%), and the number of optimal embryos was also higher (MD = 0.28, 95%CI [0.08, 0.48], *p* = 0.005, I^2^ = 94%) in women with GH co-treatment. In addition, three studies involving 299 patients observed the cycle cancellation rate according to whether or not they received GH supplementation. We noted a decrease in cycle cancellation rate in women with GH treatment (RR = 0.46, 95%CI [0.24, 0.89], *p* = 0.02, I^2^ = 0%) (**Table 2**). The sensitivity analysis verified that no individual study impacted the pooled estimates.

#### 3.4.3 Cleavage rate and miscarriage rate

However, no statistically significant difference in cleavage rate (RR = 1.12, 95%CI [0.99, 1.27], *p* = 0.07, I^2^ = 51%) and miscarriage rate (RR = 1.02, 95%CI [0.50, 2.10], *p* = 0.95, I^2^ = 0%) was detected between the GH group and the control group (**Table 2**). The results were not modified after the sensitivity analysis.

#### 3.4.4 Fertilization rate and embryo implantation rate

Five RCTs, including 665 patients, were identified to evaluate the effect of GH addition on fertilization rate. We observed a remarkable increase in the fertilization rate of infertile women receiving GH treatment (RR = 1.33, 95%CI [1.18, 1.50], *p* < 0.00001, I^2^ = 48%). In addition, three studies recruiting 430 patients reported embryo implantation rates. The meta-analysis revealed a considerable increase in the embryo implantation rate in the GH group (RR = 1.56, 95%CI [1.21, 2.01], *p* = 0.0006, I^2^ = 17%) (**Table 2**). No individual study affected the results after examination by sensitivity analysis.

#### 3.4.5 E_2_ on the day of hCG and endometrial thickness on the day of hCG

With respect to the E_2_ levels on the day of hCG, data from seven studies comprising 602 patients showed that the improvement in E_2_ levels on the day of hCG was connected with a combination of GH (SMD = 1.18, 95%CI [0.46, 1.91], *p* = 0.001, I^2^ = 94%). Meanwhile, five studies including 449 patients reported the endometrial thickness on the day of hCG. The result implied an enormous increase in the endometrial thickness on the day of hCG with the administration of GH (MD = 0.75, 95%CI [0.41, 1.09], *p* < 0.0001, I^2^ = 57%) (**Table 2**). The sensitivity analysis proved that our results were robust.

#### 3.4.6 Total days of Gn used and total dose of Gn used

Six studies focused on the total days of Gn used. After omitting one study (14) via sensitivity analysis, the heterogeneity decreased from 93% to 42%. The pooled result from five studies involving 438 patients presented a significant reduction in the total days of Gn used in the GH group (MD = -0.26, 95%CI [-0.46, -0.06], *p* = 0.01, I^2^ = 42%). Additionally, six studies provided sufficient data to compare the total dose of Gn used in women with GH treatment and those without GH treatment. Pooling of these data indicated a striking association between GH treatment and the total dose of Gn used (MD = -460.97, 95%CI [-617.20, -304.73], *p* < 0.00001, I^2^ = 90%) (**Table 2**).

### 3.5 Subgroup analysis

Given the three pooled results on the number of oocytes retrieved, the number of optimal embryos, and the level of E_2_ on the day of hCG were detected with substantial heterogeneity (I^2^ > 60%), subgroup analysis was performed according to GH dose and GnRH protocol during ovarian stimulation. However, when comparing the association of different GnRH protocols with the number of optimal embryos and level of E_2_ on the day of hCG, only one study reported available data on GnRH agonist protocol. Therefore, we conducted subgroup analysis according to GH dose for the two outcomes only.

#### 3.5.1 Number of oocytes retrieved

In the subgroup analysis for GH dose, there was no statistical difference in the outcome of the number of oocytes retrieved between women with GH dose ≥4.5 IU/d treatment and GH dose <4.5 IU/d treatment. However, the result showed that the GnRH antagonist protocol combined with GH supplementation was more favorable in improving the number of oocytes retrieved compared to the GnRH agonist protocol (MD = 1.09, 95%CI [0.59, 1.58], *p* < 0.0001, I^2^ = 90%) (**Table 3**).

**Table 3:** The subgroup analysis of the correlation of GH with clinical outcomes.

Subgroup	Study (n)	Case (n)	SMD/MD 95% CI	*p*	I^2^ (%)	Model
Number of oocytes retrieved						
GH dose ≥ 4.5 IU/d	4	368	1.38 [0.88, 1.87]	<0.00001	61	Random
GH dose < 4.5 IU/d	3	218	0.67 [0.18, 1.15]	0.008	85	Random
GnRH antagonist protocol	6	481	1.09 [0.59, 1.58]	<0.0001	90	Random
GnRH agonist protocol	2	225	0.36 [-0.25, 0.96]	0.25	65	Random
Number of optimal embryos						
GH dose ≥ 4.5 IU/d	2	205	0.31 [0.01, 0.62]	0.04	73	Random
GH dose < 4.5 IU/d	2	170	0.25 [-0.11, 0.61]	0.17	96	Random
E_2_ on the day of hCG						
GH dose ≥ 4.5 IU/d	4	368	1.25 [0.35, 2.15]	0.007	94	Random
GH dose < 4.5 IU/d	3	234	1.10 [-0.34, 2.54]	0.14	96	Random

#### 3.5.2 Number of optimal embryos and level of E_2_ on the day of hCG

Subgroup analysis performed according to the GH dose indicated a statistically significant difference between the two outcomes. The number of optimal embryos (MD = 0.31, 95%CI [0.01, 0.62], *p* = 0.04, I^2^ = 73%) and the level of E_2_ on the day of hCG (SMD = 1.25, 95%CI [0.35, 2.15], *p* = 0.007, I^2^ = 94%) were remarkably increased when GH dose ≥4.5 IU/d was adopted, whereas trials with GH dose < 4.5 IU/d showed uncertain efficacy (**Table 3**).

## 4 Discussion

Infertility is a widespread reproductive health problem globally. Approximately 12.7% of reproductive-age females suffered from infertility in the US, and 25% of cases of infertility were diagnosed as ovulatory disorders (2). One of the major causes of infertility is DOR (2). Nevertheless, the etiology of DOR is uncertain due to the various complicated factors involved. Abundant literature has elucidated the association of DOR with some pathogenic factors, including environmental influences (triclosan and arsenic exposure, per- and polyfluoroalkyl substances intake) (26–28), unhealthy habits (cigarette, alcohol, and addictive drug consumption) (29), medical complications (gynecological surgery, radiotherapy, and chemotherapy) (30, 31), maternal exposures (intrauterine nutrition restriction, chronic gestational hypoxia) (32, 33), autoimmune diseases (HIV infection, thyroid hormone imbalance) (34, 35), and so forth. Notably, the deterioration of gamete quality and the progressive loss of oocytes occur naturally with older age. Therefore, a burgeoning number of women with DOR are seeking ART treatment for infertility yearly (36). Despite recent advances in ART, the outcomes of natural cycle IVF for DOR are unsatisfying due to the high cycle cancellation rate. For this reason, diverse stimulation interventions and protocols have been recommended to ameliorate IVF outcomes in infertile women with DOR. These schemes used for ovulation induction include the addition of adjuvant therapy, increased gonadotropin dosages, and different procedures for pituitary suppression during ovarian stimulation (37–39). However, high doses of gonadotrophins are often associated with a range of unexpected side effects (40). Hence, exploring an optimal adjuvant treatment remains an ongoing clinical challenge.

GH treatment in conjunction with ovarian stimulation has been considered an efficient strategy for enhancing the outcomes of IVF in infertile women (41). The molecular mechanisms of GH in the reproductive field are rather complex. Substantial evidence has demonstrated that GH could improve oocyte developmental competence by alleviating apoptosis and elevating mitochondrial membrane potential through activating the PI3K-AKT and the Sirt3-Sod2 signaling pathway in granulosa cells; meanwhile, GH may also promote cell proliferation and participate in the steroidogenic process via PI3K-AKT and PLC-PKC signaling to interact with LHR and FSHR and then regulate the functions of granulosa cells and the follicle development (42). Additionally, Liu et al., using *in vitro* and *in vivo* experiments, revealed that GH could significantly ameliorate the decline of oocyte quality and depletion of ovarian reserve associated with advanced age via decreasing the expression of γH2AX and inhibiting Fos and Jun signaling pathways in oocytes (43). In terms of uterine receptivity, GH may mediate endometrial thickness and increase endometrial blood perfusion by upregulating IGF-1 and VEGF together with ITGB3 in the uterus (44, 45).

A previous meta-analysis (46) focusing on GH for patients with poor ovarian responders undergoing IVF suggested that GH is beneficial for women in increasing the number of oocytes retrieved and MII oocytes, along with the clinical pregnancy rate and the number of embryos available to transfer. However, they failed to estimate the association of GH supplementation with cycle cancellation rate, cleavage rate, fertilization rate, E_2_ levels on the day of hCG, endometrial thickness on the day of hCG, total days of Gn used, and total dose of Gn applied. Furthermore, the previous study didn’t distinguish the effects of GH for women with different ovarian reserves. Therefore, to provide a more specific reflection on the significance of GH in a unique population, we focused our study on women with DOR.

In this systematic review, we estimated a potential association of GH treatment in infertile women with DOR undergoing IVF to provide evidence for informing clinical practice. Our research extended and replicated former reviews on the effectiveness of GH in fertility, which, however, generally focused on poor responders. According to our meta-analysis, adjuvant GH not only increased the clinical pregnancy rate, the number of oocytes retrieved, the optimal embryos rate, and the number of optimal embryos but also decreased the cycle cancellation rate of infertile women with DOR, which was the most critical outcome for women undergoing IVF. Moreover, GH co-treatment was more beneficial in improving the fertilization rate and embryo implantation rate. In particular, the administration of GH resulted in an obvious increase in the E_2_ levels and endometrial thickness on the day of hCG, which may be a novel therapeutic option for women with a thin endometrium. Similarly, a significant decrease in the total days and dose of gonadotropin applied was observed when GH was added during ovarian stimulation. Based on subgroup analyses, we also noted there was no dose-dependent connection between adjuvant GH and the number of oocytes retrieved, whereas an increased dose of GH (≥4.5 IU/d) was superior in improving the number of optimal embryos and E_2_ levels on the day of hCG. Furthermore, the combination of the GnRH antagonist protocol with GH significantly promoted the number of oocytes retrieved compared to the GnRH agonist protocol.

To the best of our knowledge, this is the first systematic review and meta-analysis evaluating the impact of GH on the outcomes in infertile women with DOR undergoing IVF. Some limitations ought to be noted in the interpretation of our findings. First, although 10 RCTs were included in this analysis, two studies (20, 24) adopted the median (25th percentile, 75th percentile) for continuous variables and one study (14) just reported the percentages without detailed data on clinical pregnancy rate and cleavage rate. As a result, we were unable to integrate the corresponding results into our analysis. The second limitation is that the ovarian stimulation protocols varied in each study, including GnRH antagonist protocol, minimal stimulation protocol, long GnRH agonist protocol, and short GnRH agonist protocol. Therefore, we were forced to divide these protocols into GnRH antagonist protocol and GnRH agonist protocol for subgroup analysis. For this reason, we could not estimate the correlation of GH plus different ovarian stimulation protocols with the outcomes of IVF. The third limitation is that all studies incorporated poor descriptions of their methodologies. For example, they did not report blinding of the participants, investigators, and assessors throughout the trial, which substantially impaired the strength of the evidence. However, since study populations are diverse in the real world of clinical practice, non-blinded pragmatic studies have been proposed to obtain clinically relevant outcomes (47). The fourth limitation is that publication bias cannot be evaluated by Begg’s and Egger’s tests, as less than ten studies were included in each outcome. Nevertheless, to accommodate this limitation, we conducted a sensitivity analysis for each estimate, and it verified that our results were robust. The fifth limitation is that the age of patients is believed to lead to differences in IVF efficacy, with younger infertile women with DOR possibly achieving greater improvement. Unfortunately, none of the studies reported outcomes based on different ages. Therefore, we can’t perform subgroup analysis to compare the clinical significance of GH co-treatment among different age groups. Lastly, our meta-analysis focused on GH in infertile women with DOR undergoing IVF. Hence, our pooled results may not be appropriate for those not diagnosed with DOR.

## 5 Conclusion

In conclusion, GH supplementation has significant clinical efficacy in improving the outcomes in infertile women with DOR undergoing IVF, including increasing the clinical pregnancy rate, number of oocytes retrieved, optimal embryos rate and number of optimal embryos, fertilization rate, embryo implantation rate, E_2_ levels, and endometrial thickness on the day of hCG, along with decreasing cycle cancellation rate, total days, and total dose of gonadotropin applied. However, our findings are insufficient to offer clinical recommendations due to the high risk of bias and heterogeneity. Therefore, well-designed studies are needed to certify our results in the future.

## Data availability statement

The original contributions presented in the study are included in the article. Further inquiries can be directed to the corresponding author.

## Author contributions

GYL and LWX designed the study. GYL implemented the methods and conducted the experiments. GYL, XFZ, and SNL analyzed and presented the results. GYL completed an initial draft. All authors contributed to the article and approved the submitted version.
